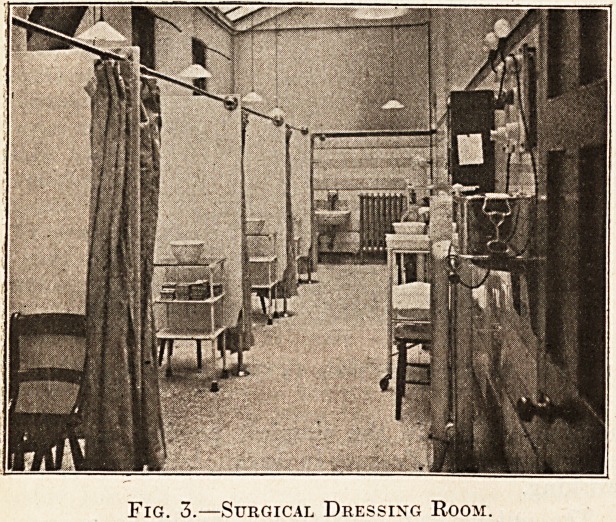# The Units of General Hospital Construction

**Published:** 1907-06-22

**Authors:** 


### HOSPITAL ADMINISTRATION.

### CONSTRUCTION AND ECONOMICS.

### THE UNITS OF GENERAL HOSPITAL CONSTRUCTION.

#### THE OUT-PATIENT UNIT.

The advantages of having an out-patient department attached to a general hospital are universally recognised, and this department holds a most important place in every modern institution. It forms a convenient and suitable channel through which patients may enter the hospital for indoor treatment. Many patients can be treated in the out-patient department without being admitted into the wards, while others may be kept under observation until their condition calls for indoor treatment.

In the out-patient department of a teaching hospital students have an opportunity of studying the more common ailments, which they will be called upon most frequently to treat when they go into practice, but which they never see in the wards. It also forms an excellent school for young physicians and surgeons, as they come into direct touch with students who are observant and critical. It should also constitute part of every nurse's training, for the experience acquired in this department is of the utmost value to a nurse, more especially if she takes up district nursing among the poor.

#### Planning the Department.

In designing an out-patient department, the position and character of the site and its relation to the hospital proper, must receive careful study. Taken in conjunction with this, the number of patients to

Fig. 1.—Waiting-hall for Out-patients.

be treated each day of the week, the number of new patients and the number of return cases in daily attendance, the number to be treated in each separate department, and the number of students in attendance must be ascertained. With these data the architect should provide the necessary

Fig. 2.—Entrance-door to Consulting Room.

accommodation in the simplest possible way, to ensure not only efficiency in design, but economy in working.

The out-patient department should be a one-story building quite apart from the hospital, but they may be connected by a covered way or corridor. The entrance should be separate and distinct from the exit and from those of the hospital proper, so that out-patients have no means of communicating with indoor convalescent patients who may be airing or exercising in the grounds. This is of the utmost importance, as infectious disease might conceivably be communicated to in-patients, or food, liquor, etc., might be handed to convalescent patients, and they in turn might pass it on to acute cases in the wards, and in this way do incalculable mischief. A separate entrance should also be provided for students.

#### The Preliminaries of Admission.

When a patient arrives at the entrance gate he should be met in the vestibule by a porter, and be directed into the inquiry room, where the registrar takes a note of the following particulars:—

Name, age, occupation, residence, employer, weekly wage, and any other details which may be considered necessary for excluding unsuitable cases.

The patient is then furnished with a numbered book or report sheet bearing his name and number, also the name of the physician, surgeon, or specialist, as the case may be, to whom he is allocated. This slip or card should have printed in bold letters instructions as to the days and hours patients should return for further treatment. It is usual to have these books or report sheets of different colours, in order that the attendant may distinguish at a glance the patients of each surgeon or physician.

Adjoining this inquiry hall two rooms should be placed, one where the patient may be examined by the resident physician, in order that he may determine to which department the patient should be sent; and another small room alongside it where any patient suspected to be suffering from a contagious or infectious disease can be retained until the case has been diagnosed, and, if it proves to be infectious, until arrangements have been made for the removal of the patient to the hospital for infectious disease. This room should have a door opening to an outside court, so that the patient can be removed to the ambulance wagon without again passing through any part of the building.

Fig. 3.—Surgical Dressing Room.

### The Out-Patients' Hall.

All patients on their second or subsequent visits to the department are passed by the porter into the large waiting hall, and are seated on benches opposite the consulting room of their medical attendant. Finger-posts with the name of the physician or surgeon are attached to each series of benches. The size of the waiting hall will be determined by the number of daily applicants, but in calculating this space it should be kept in mind that many of the patients are children, cripples or physically enfeebled, and as they are invariably accompanied by one, and sometimes two, relatives, the waiting hall should seat at least double the average number of patients applying for daily treatment. A waiting hall 80 ft. by 30 ft. will give accommodation for over 400 patients and still leave ample room for dividing the patients for the various departments. The arrangement of the waiting hall requires careful study, not only for convenience in working, but for economy in service and easy and effective supervision.

### Lighting Arrangements.

If possible, the building should be so designed that the consulting rooms get the maximum of north light. Adjoining the waiting halls suitable lavatory accommodation should be provided for both sexes. These lavatories should be in an open court, with a cut-off passage completely detaching them from other parts of the building.

A convenient method of communication between the physician or surgeon and the waiting hall has been adopted in the out-patient department of the Western Infirmary, Glasgow, which avoids the necessity of a separate porter to regulate the patients for each consultant. An automatic arrangement has been installed whereby a bell is rung, and the words "next patient" are illuminated over the door leading to the consulting room when the medical officer pushes a knob. By this arrangement the patients pass into the consulting room in turn.

### Male and Female Consulting Rooms.

Two rooms should be provided for each consulting room, one for each sex, where patients may undress if necessary. These should be so placed that patients may pass from the consulting room to either dressing room without being seen from the waiting hall. In each a sink with hot and cold water is provided; also a portable light, in addition to the usual light for illuminating purposes. The building should be so arranged that patients retire from these dressing rooms without again entering the consulting room. The exit corridor should be so placed that patients may pass out without returning to the general waiting hall. This corridor ends in the dispensary waiting hall adjoining the exit vestibule.

The necessity which exists for suitable accommodation being provided for the dressing of minor surgical cases is often overlooked in designing an out-patient department. Two well-lit and well-ventilated rooms, one for each sex, should adjoin the waiting hall for the treatment of such cases.

### The Surgical Dressing Room.

Fig. 3 illustrates such a room, showing a range of stalls, each stall having a sink with hot and cold water, some of the sinks being placed on the floor level for the treatment of cases such as injuries to the feet and ulcers of the leg. The stalls are divided by a partition of enamel slate, and have washable curtains in front, so that each stall is self-contained. An electrical steriliser for instruments and stands for lotions and dressings are provided on the wall opposite the stalls. Four basins should be fitted up, two at each end of the room, for the exclusive use of the nurses and dressers. Although only minor operations are performed in an out-patient department, nothing should be left undone to secure the strictest asepsis. This is in the interest not only of the patient, but also of the education of the student.

*(To be continued.)*

## Figures and Tables

**Fig. 1. f1:**
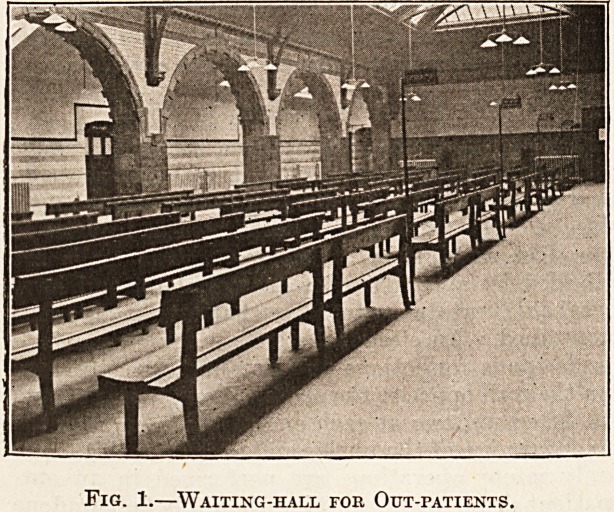


**Fig. 2. f2:**
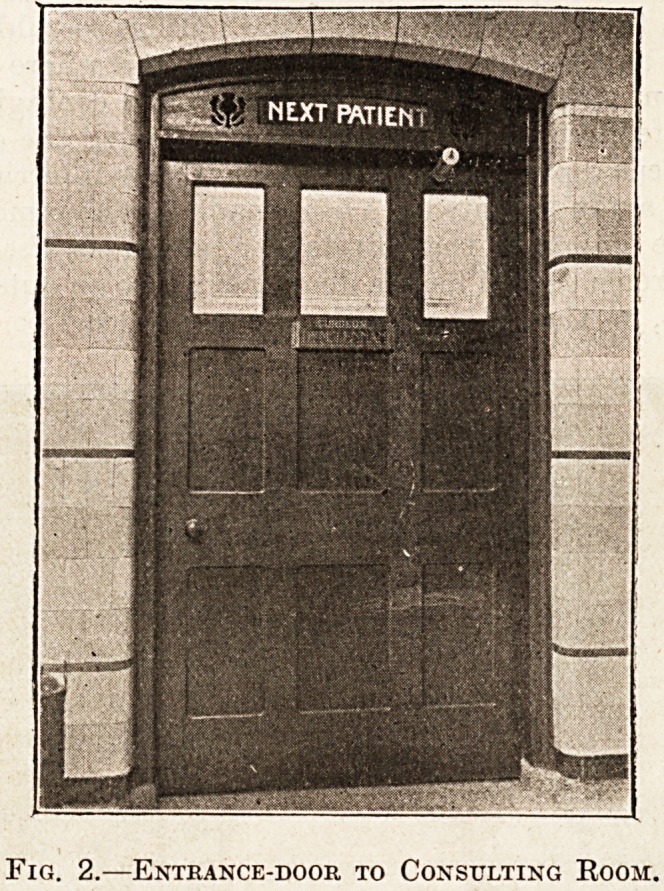


**Fig. 3. f3:**